# Dichloridobis(di-*tert*-butyl­methyl­phosphine oxide-κ*O*)diphenyl­tin(IV)

**DOI:** 10.1107/S1600536808013809

**Published:** 2008-05-10

**Authors:** Manuela Müller, Hans-Wolfram Lerner, Michael Bolte

**Affiliations:** a Institut für Anorganische Chemie, J. W. Goethe-Universität Frankfurt, Max-von-Laue-Strasse 7, 60438 Frankfurt/Main, Germany

## Abstract

The complete mol­ecule of the title compound, [Sn(C_6_H_5_)_2_Cl_2_(C_9_H_21_OP)_2_], is generated by crystallographic inversion symmetry, the Sn atom is located on a special position of site symmetry 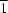. The Sn atom adopts an all-*trans* SnC_2_O_2_Cl_2_ octa­hedral geometry. As a consequence of the bulky substituents at the O atom, the P—O—Sn bond angle is 163.9 (3)°.

## Related literature

For related literature, see: Lerner *et al.* (2005 ▶); Ruth *et al.* (2005 ▶, 2007 ▶).
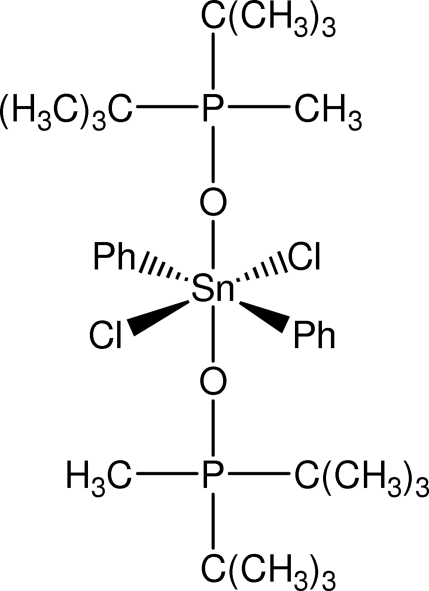


## Experimental

### 

#### Crystal data

[Sn(C_6_H_5_)_2_Cl_2_(C_9_H_21_OP)_2_]
*M* *_r_* = 696.25
Monoclinic, 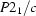

*a* = 12.1782 (19) Å
*b* = 9.0866 (8) Å
*c* = 16.339 (2) Å
β = 111.518 (11)°
*V* = 1682.0 (4) Å^3^
*Z* = 2
Mo *K*α radiation
μ = 1.04 mm^−1^
*T* = 173 (2) K
0.13 × 0.09 × 0.07 mm

#### Data collection

Stoe IPDSII two-circle diffractometer
Absorption correction: multi-scan (*MULABS*; Spek, 2003 ▶; Blessing, 1995 ▶) *T*_min_ = 0.877, *T*_max_ = 0.931
11731 measured reflections
3145 independent reflections
1754 reflections with *I* > 2σ(*I*)
*R*_int_ = 0.087

#### Refinement

*R*[*F* ^2^ > 2σ(*F* ^2^)] = 0.058
*wR*(*F* ^2^) = 0.098
*S* = 0.88
3145 reflections
169 parameters
H-atom parameters constrained
Δρ_max_ = 0.44 e Å^−3^
Δρ_min_ = −0.83 e Å^−3^

### 

Data collection: *X-AREA* (Stoe & Cie, 2001 ▶); cell refinement: *X-AREA*; data reduction: *X-AREA*; program(s) used to solve structure: *SHELXS97* (Sheldrick, 2008 ▶); program(s) used to refine structure: *SHELXL97* (Sheldrick, 2008 ▶); molecular graphics: *XP* in *SHELXTL-Plus* (Sheldrick, 2008 ▶); software used to prepare material for publication: *SHELXL97* and *PLATON* (Spek, 2003 ▶).

## Supplementary Material

Crystal structure: contains datablocks I, global. DOI: 10.1107/S1600536808013809/hb2731sup1.cif

Structure factors: contains datablocks I. DOI: 10.1107/S1600536808013809/hb2731Isup2.hkl

Additional supplementary materials:  crystallographic information; 3D view; checkCIF report

## Figures and Tables

**Table 1:** Selected bond lengths (Å)

Sn1—C41	2.128 (7)
Sn1—O1	2.232 (4)
Sn1—Cl1	2.5567 (16)

**Fig. 1.**
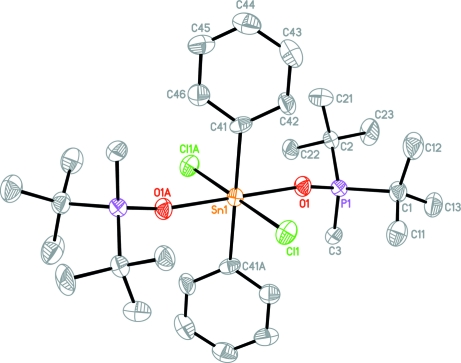
Perspective view of (I) with displacement ellipsoids drawn at the 50% probability level; H atoms are omitted for clarity. Symmetry operator for generating equivalent atoms: (A) 1 - x, 1 - y, 1 - z.

## supplementary crystallographic information

### Comment

We are interested in Lewis-acidic Sn(IV) compounds and their reactivity towards Lewis bases. Recently we have reported the synthesis and structure of {Zn[Sn(CH_2_SMe)_4_]0.5Cl_2_}_n_ and Sn(CH_2_PPh_2_)_4_ (Ruth *et al.*, 2007). In contrast to [SnCl_4_].[CH_3_SCH_3_]_2_ which forms an adduct in solid state with a six-coordinated Sn atom (Ruth *et al.*, 2005), the Sn(IV) centers in {Zn[Sn(CH_2_SMe)_4_]0.5Cl_2_}_n_ and Sn(CH_2_PPh_2_)_4_ are tetra-coordinated. However, Me_3_SnCl forms an adduct with Me_3_SnOH and H_2_O in which the Sn atoms possess the coordination number five. It is interesting to note that this adduct represents an intermediate in Me_3_SnCl hydrolysis (Lerner *et al.*, 2005). We report here the X-ray crystal structure analysis of the title adduct [Ph_2_SnCl_2_].[*t*Bu_2_MePO]_2_, (I). The synthesis of (I) was achieved by treatment of Ph_2_SnCl_2_ with two equivalents of *t*Bu_2_MePO as indicated in the equation below.

Compound (I) has crystallographic inversion symmetry with just half a molecule in the asymmetric unit. The Sn atom is hexacoordinated by three pairs of different ligands in an octahedral fashion (Table 1). All ligand pairs of the same kind are mutually *trans* at the Sn atom (Fig. 1). As a consequence of the bulky substituents at the O atom the P—O—Sn angle is enlarged to 163.9 (3)°.

### Experimental

*t*Bu_2_MePO (2.05 mmol) was added with stirring at ambient temperature to a solution of Ph_2_SnCl_2_ (0.58 mmol) in 25 ml THF. Colourless blocks of (I) were grown by storing this solution at room temperature for several weeks.

### Refinement

The H atoms were geometrically positioned (C—H = 0.95-0.98Å) and refined as riding with U_iso_(H) = 1.2*U*_eq_(C) or 1.5 *U*_eq_(methyl C).

### Crystal data

**Table:**

[Sn(C_6_H_5_)_2_Cl_2_(C_9_H_21_OP)_2_]	*F*(000) = 724
*M**_r_* = 696.25	*D*_x_ = 1.375 Mg m^−^^3^
Monoclinic, *P*2_1_/*c*	Mo *K*α radiation, λ = 0.71073 Å
Hall symbol: -P 2ybc	Cell parameters from 4261 reflections
*a* = 12.1782 (19) Å	θ = 3.5–25.4°
*b* = 9.0866 (8) Å	µ = 1.04 mm^−^^1^
*c* = 16.339 (2) Å	*T* = 173 K
β = 111.518 (11)°	Block, colourless
*V* = 1682.0 (4) Å^3^	0.13 × 0.09 × 0.07 mm
*Z* = 2	

### Data collection

**Table:**

Stoe IPDSII two-circle diffractometer	3145 independent reflections
Radiation source: fine-focus sealed tube	1754 reflections with *I* > 2σ(*I*)
graphite	*R*_int_ = 0.087
ω scans	θ_max_ = 25.6°, θ_min_ = 3.4°
Absorption correction: multi-scan (*MULABS*; Spek, 2003; Blessing, 1995)	*h* = −14→14
*T*_min_ = 0.877, *T*_max_ = 0.931	*k* = −10→11
11731 measured reflections	*l* = −19→19

### Refinement

**Table:**

Refinement on *F*^2^	Primary atom site location: structure-invariant direct methods
Least-squares matrix: full	Secondary atom site location: difference Fourier map
*R*[*F*^2^ > 2σ(*F*^2^)] = 0.058	Hydrogen site location: inferred from neighbouring sites
*wR*(*F*^2^) = 0.098	H-atom parameters constrained
*S* = 0.88	*w* = 1/[σ^2^(*F*_o_^2^) + (0.0102*P*)^2^] where *P* = (*F*_o_^2^ + 2*F*_c_^2^)/3
3145 reflections	(Δ/σ)_max_ < 0.001
169 parameters	Δρ_max_ = 0.44 e Å^−^^3^
0 restraints	Δρ_min_ = −0.83 e Å^−^^3^

### Special details

**Table:**

Geometry. All e.s.d.'s (except the e.s.d. in the dihedral angle between two l.s. planes) are estimated using the full covariance matrix. The cell e.s.d.'s are taken into account individually in the estimation of e.s.d.'s in distances, angles and torsion angles; correlations between e.s.d.'s in cell parameters are only used when they are defined by crystal symmetry. An approximate (isotropic) treatment of cell e.s.d.'s is used for estimating e.s.d.'s involving l.s. planes.
Refinement. Refinement of *F*^2^ against ALL reflections. The weighted *R*-factor *wR* and goodness of fit *S* are based on *F*^2^, conventional *R*-factors *R* are based on *F*, with *F* set to zero for negative *F*^2^. The threshold expression of *F*^2^ > σ(*F*^2^) is used only for calculating *R*-factors(gt) *etc*. and is not relevant to the choice of reflections for refinement. *R*-factors based on *F*^2^ are statistically about twice as large as those based on *F*, and *R*- factors based on ALL data will be even larger.

### Fractional atomic coordinates and isotropic or equivalent isotropic displacement parameters (Å^2^)

**Table:**

	*x*	*y*	*z*	*U*_iso_*/*U*_eq_	
Sn1	0.5000	0.5000	0.5000	0.02649 (18)	
Cl1	0.46725 (18)	0.3097 (2)	0.60405 (11)	0.0361 (5)	
P1	0.31774 (17)	0.7748 (2)	0.56073 (11)	0.0271 (4)	
O1	0.3737 (4)	0.6468 (5)	0.5312 (3)	0.0300 (11)	
C1	0.2236 (7)	0.7021 (8)	0.6188 (4)	0.0340 (17)	
C2	0.2381 (6)	0.8878 (8)	0.4633 (4)	0.0337 (17)	
C3	0.4234 (7)	0.8947 (8)	0.6369 (4)	0.0360 (18)	
H3A	0.4684	0.8398	0.6903	0.054*	
H3B	0.3822	0.9768	0.6521	0.054*	
H3C	0.4774	0.9331	0.6100	0.054*	
C11	0.3017 (7)	0.5921 (9)	0.6885 (5)	0.043 (2)	
H11A	0.3721	0.6430	0.7282	0.064*	
H11B	0.3257	0.5114	0.6589	0.064*	
H11C	0.2568	0.5523	0.7225	0.064*	
C12	0.1142 (8)	0.6241 (10)	0.5567 (5)	0.058 (3)	
H12A	0.0656	0.6940	0.5126	0.086*	
H12B	0.0686	0.5850	0.5903	0.086*	
H12C	0.1380	0.5431	0.5273	0.086*	
C13	0.1867 (8)	0.8238 (9)	0.6696 (5)	0.045 (2)	
H13A	0.2574	0.8731	0.7101	0.068*	
H13B	0.1428	0.7798	0.7031	0.068*	
H13C	0.1366	0.8958	0.6279	0.068*	
C21	0.1735 (8)	0.7857 (10)	0.3860 (5)	0.054 (2)	
H21A	0.1137	0.7289	0.3994	0.081*	
H21B	0.2303	0.7182	0.3762	0.081*	
H21C	0.1351	0.8446	0.3329	0.081*	
C22	0.3310 (6)	0.9731 (9)	0.4389 (4)	0.036 (2)	
H22A	0.3744	1.0397	0.4871	0.054*	
H22B	0.2916	1.0304	0.3853	0.054*	
H22C	0.3862	0.9035	0.4288	0.054*	
C23	0.1515 (7)	0.9993 (15)	0.4782 (5)	0.064 (3)	
H23A	0.0918	0.9463	0.4938	0.096*	
H23B	0.1128	1.0558	0.4242	0.096*	
H23C	0.1950	1.0664	0.5261	0.096*	
C41	0.3564 (7)	0.4098 (7)	0.3934 (4)	0.0276 (16)	
C42	0.2454 (7)	0.3792 (8)	0.3993 (5)	0.0368 (18)	
H42	0.2346	0.3983	0.4530	0.044*	
C43	0.1523 (8)	0.3224 (9)	0.3291 (5)	0.049 (2)	
H43	0.0771	0.3099	0.3334	0.059*	
C44	0.1696 (8)	0.2832 (9)	0.2512 (5)	0.046 (2)	
H44	0.1076	0.2388	0.2039	0.055*	
C45	0.2763 (8)	0.3097 (9)	0.2440 (4)	0.042 (2)	
H45	0.2876	0.2845	0.1912	0.051*	
C46	0.3695 (7)	0.3738 (8)	0.3140 (4)	0.0347 (18)	
H46	0.4424	0.3930	0.3074	0.042*	

### Atomic displacement parameters (Å^2^)

**Table:**

	*U*^11^	*U*^22^	*U*^33^	*U*^12^	*U*^13^	*U*^23^
Sn1	0.0255 (4)	0.0305 (4)	0.0254 (3)	0.0007 (6)	0.0116 (3)	−0.0013 (5)
Cl1	0.0442 (12)	0.0352 (11)	0.0335 (9)	−0.0015 (10)	0.0198 (9)	0.0010 (8)
P1	0.0226 (11)	0.0324 (11)	0.0262 (8)	0.0000 (9)	0.0086 (8)	−0.0023 (8)
O1	0.031 (3)	0.029 (3)	0.033 (2)	0.006 (2)	0.015 (2)	−0.002 (2)
C1	0.032 (5)	0.037 (4)	0.036 (4)	−0.005 (4)	0.017 (3)	−0.010 (3)
C2	0.024 (4)	0.045 (5)	0.034 (4)	0.001 (4)	0.013 (3)	−0.003 (3)
C3	0.032 (5)	0.052 (5)	0.025 (3)	−0.003 (4)	0.012 (3)	0.000 (3)
C11	0.051 (6)	0.039 (5)	0.046 (4)	−0.005 (4)	0.027 (4)	0.004 (4)
C12	0.055 (6)	0.072 (7)	0.058 (5)	−0.030 (5)	0.034 (5)	−0.027 (5)
C13	0.048 (6)	0.052 (5)	0.046 (4)	−0.006 (5)	0.029 (4)	−0.003 (4)
C21	0.038 (5)	0.078 (7)	0.036 (4)	0.003 (5)	0.002 (4)	0.001 (4)
C22	0.034 (4)	0.040 (6)	0.039 (3)	0.015 (4)	0.018 (3)	0.009 (4)
C23	0.057 (6)	0.087 (6)	0.059 (4)	0.056 (8)	0.033 (4)	0.026 (7)
C41	0.036 (5)	0.021 (4)	0.021 (3)	0.000 (3)	0.004 (3)	−0.004 (3)
C42	0.024 (4)	0.047 (5)	0.043 (4)	−0.009 (4)	0.017 (4)	−0.013 (4)
C43	0.033 (5)	0.052 (6)	0.066 (5)	0.000 (5)	0.022 (4)	0.000 (4)
C44	0.036 (5)	0.041 (5)	0.048 (4)	−0.002 (4)	0.003 (4)	−0.006 (4)
C45	0.043 (5)	0.047 (5)	0.029 (4)	−0.004 (4)	0.004 (4)	0.001 (3)
C46	0.036 (5)	0.032 (5)	0.035 (4)	0.007 (4)	0.012 (4)	0.008 (3)

### Geometric parameters (Å, °)

**Table:**

Sn1—C41^i^	2.128 (7)	C12—H12C	0.9800
Sn1—C41	2.128 (7)	C13—H13A	0.9800
Sn1—O1	2.232 (4)	C13—H13B	0.9800
Sn1—O1^i^	2.232 (4)	C13—H13C	0.9800
Sn1—Cl1^i^	2.5567 (16)	C21—H21A	0.9800
Sn1—Cl1	2.5567 (16)	C21—H21B	0.9800
P1—O1	1.513 (4)	C21—H21C	0.9800
P1—C3	1.794 (7)	C22—H22A	0.9800
P1—C2	1.842 (7)	C22—H22B	0.9800
P1—C1	1.858 (7)	C22—H22C	0.9800
C1—C12	1.522 (10)	C23—H23A	0.9800
C1—C11	1.552 (10)	C23—H23B	0.9800
C1—C13	1.545 (9)	C23—H23C	0.9800
C2—C22	1.541 (10)	C41—C46	1.402 (8)
C2—C21	1.533 (11)	C41—C42	1.418 (10)
C2—C23	1.544 (11)	C42—C43	1.383 (10)
C3—H3A	0.9800	C42—H42	0.9500
C3—H3B	0.9800	C43—C44	1.412 (10)
C3—H3C	0.9800	C43—H43	0.9500
C11—H11A	0.9800	C44—C45	1.367 (11)
C11—H11B	0.9800	C44—H44	0.9500
C11—H11C	0.9800	C45—C46	1.409 (10)
C12—H12A	0.9800	C45—H45	0.9500
C12—H12B	0.9800	C46—H46	0.9500
			
C41^i^—Sn1—C41	180.0	C1—C12—H12B	109.5
C41^i^—Sn1—O1	90.6 (2)	H12A—C12—H12B	109.5
C41—Sn1—O1	89.4 (2)	C1—C12—H12C	109.5
C41^i^—Sn1—O1^i^	89.4 (2)	H12A—C12—H12C	109.5
C41—Sn1—O1^i^	90.6 (2)	H12B—C12—H12C	109.5
O1—Sn1—O1^i^	180.0	C1—C13—H13A	109.5
C41^i^—Sn1—Cl1^i^	90.10 (18)	C1—C13—H13B	109.5
C41—Sn1—Cl1^i^	89.90 (18)	H13A—C13—H13B	109.5
O1—Sn1—Cl1^i^	92.09 (12)	C1—C13—H13C	109.5
O1^i^—Sn1—Cl1^i^	87.91 (12)	H13A—C13—H13C	109.5
C41^i^—Sn1—Cl1	89.90 (18)	H13B—C13—H13C	109.5
C41—Sn1—Cl1	90.10 (18)	C2—C21—H21A	109.5
O1—Sn1—Cl1	87.91 (12)	C2—C21—H21B	109.5
O1^i^—Sn1—Cl1	92.09 (12)	H21A—C21—H21B	109.5
Cl1^i^—Sn1—Cl1	180.0	C2—C21—H21C	109.5
O1—P1—C3	113.3 (3)	H21A—C21—H21C	109.5
O1—P1—C2	108.0 (3)	H21B—C21—H21C	109.5
C3—P1—C2	106.1 (3)	C2—C22—H22A	109.5
O1—P1—C1	108.9 (3)	C2—C22—H22B	109.5
C3—P1—C1	106.4 (3)	H22A—C22—H22B	109.5
C2—P1—C1	114.3 (3)	C2—C22—H22C	109.5
P1—O1—Sn1	163.9 (3)	H22A—C22—H22C	109.5
C12—C1—C11	109.9 (7)	H22B—C22—H22C	109.5
C12—C1—C13	109.6 (6)	C2—C23—H23A	109.5
C11—C1—C13	106.7 (6)	C2—C23—H23B	109.5
C12—C1—P1	112.3 (4)	H23A—C23—H23B	109.5
C11—C1—P1	106.1 (5)	C2—C23—H23C	109.5
C13—C1—P1	112.0 (5)	H23A—C23—H23C	109.5
C22—C2—C21	107.1 (6)	H23B—C23—H23C	109.5
C22—C2—C23	108.7 (7)	C46—C41—C42	116.8 (7)
C21—C2—C23	110.7 (7)	C46—C41—Sn1	120.6 (5)
C22—C2—P1	107.5 (5)	C42—C41—Sn1	122.6 (5)
C21—C2—P1	108.9 (5)	C43—C42—C41	122.0 (6)
C23—C2—P1	113.8 (4)	C43—C42—H42	119.0
P1—C3—H3A	109.5	C41—C42—H42	119.0
P1—C3—H3B	109.5	C42—C43—C44	119.6 (7)
H3A—C3—H3B	109.5	C42—C43—H43	120.2
P1—C3—H3C	109.5	C44—C43—H43	120.2
H3A—C3—H3C	109.5	C45—C44—C43	119.6 (8)
H3B—C3—H3C	109.5	C45—C44—H44	120.2
C1—C11—H11A	109.5	C43—C44—H44	120.2
C1—C11—H11B	109.5	C44—C45—C46	120.7 (7)
H11A—C11—H11B	109.5	C44—C45—H45	119.6
C1—C11—H11C	109.5	C46—C45—H45	119.6
H11A—C11—H11C	109.5	C41—C46—C45	121.1 (7)
H11B—C11—H11C	109.5	C41—C46—H46	119.4
C1—C12—H12A	109.5	C45—C46—H46	119.4
			
C3—P1—O1—Sn1	−21.3 (11)	C1—P1—C2—C21	−79.8 (6)
C2—P1—O1—Sn1	95.9 (10)	O1—P1—C2—C23	165.5 (7)
C1—P1—O1—Sn1	−139.5 (9)	C3—P1—C2—C23	−72.7 (7)
C41^i^—Sn1—O1—P1	31.6 (10)	C1—P1—C2—C23	44.2 (8)
C41—Sn1—O1—P1	−148.4 (10)	O1—Sn1—C41—C46	142.0 (5)
Cl1^i^—Sn1—O1—P1	−58.5 (10)	O1^i^—Sn1—C41—C46	−38.0 (5)
Cl1—Sn1—O1—P1	121.5 (10)	Cl1^i^—Sn1—C41—C46	49.9 (5)
O1—P1—C1—C12	−69.5 (6)	Cl1—Sn1—C41—C46	−130.1 (5)
C3—P1—C1—C12	168.1 (6)	O1—Sn1—C41—C42	−39.9 (6)
C2—P1—C1—C12	51.4 (7)	O1^i^—Sn1—C41—C42	140.1 (6)
O1—P1—C1—C11	50.5 (5)	Cl1^i^—Sn1—C41—C42	−132.0 (6)
C3—P1—C1—C11	−71.9 (5)	Cl1—Sn1—C41—C42	48.0 (6)
C2—P1—C1—C11	171.4 (5)	C46—C41—C42—C43	−2.7 (11)
O1—P1—C1—C13	166.6 (5)	Sn1—C41—C42—C43	179.1 (6)
C3—P1—C1—C13	44.2 (6)	C41—C42—C43—C44	4.7 (12)
C2—P1—C1—C13	−72.5 (6)	C42—C43—C44—C45	−3.8 (12)
O1—P1—C2—C22	−74.1 (5)	C43—C44—C45—C46	0.8 (12)
C3—P1—C2—C22	47.7 (5)	C42—C41—C46—C45	−0.3 (10)
C1—P1—C2—C22	164.5 (5)	Sn1—C41—C46—C45	178.0 (6)
O1—P1—C2—C21	41.6 (6)	C44—C45—C46—C41	1.2 (12)
C3—P1—C2—C21	163.4 (5)		

Symmetry codes: (i) −*x*+1, −*y*+1, −*z*+1.
